# Investigation of Toll-Like Receptor-2 (2258G/A) and Interferon Gamma (+874T/A) Gene Polymorphisms among Infertile Women with Female Genital Tuberculosis

**DOI:** 10.1371/journal.pone.0130273

**Published:** 2015-06-26

**Authors:** Venkanna Bhanothu, Vemu Lakshmi, Jane P. Theophilus, Roya Rozati, Prabhakar Badhini, Boda Vijayalaxmi

**Affiliations:** 1 Department of Zoology, University College of Science, Osmania University, Hyderabad, Telangana State, India; 2 Department of Microbiology, Nizam’s Institute of Medical Sciences, Hyderabad, Telangana State, India; 3 Department of Obstetrics and Gynecology, Owaisi Hospital & Research Centre, Hyderabad, Telangana State, India; 4 Department of Genetics, University College of Science, Osmania University, Hyderabad, Telangana State, India; Nanjing Medical University, CHINA

## Abstract

**Background:**

Toll-like receptor 2 (TLR2) and interferon-gamma (IFN-γ) coordinate with a diverse array of cellular programs through the transcriptional regulation of immunologically relevant genes and play an important role in immune system, reproductive physiology and basic pathology. Alterations in the functions of TLR2 2258G (guanine)/ A, IFN-γ (+874T/A) and signalling molecules that result from polymorphisms are often associated with susceptibility or resistance, which may, in turn, establish the innate host response to various infectious diseases. Presently, we proposed to investigate the risk of common single nucleotide polymorphism (SNP) of TLR2 and IFN-γ genes, for their effect on infertility in women with female genital tuberculosis (FGTB) and healthy women as controls.

**Methodology/Principal Findings:**

Genotyping of TLR2 and IFN-γ gene polymorphisms was performed by amplification refractory mutation system multi-gene/multi-primer polymerase chain reaction followed by restriction fragment length polymorphism in 175 FGTB patients and 100 healthy control women (HCW). The TLR2 polymorphism [adenine (A) allele] was observed in 57.7 and 58.0% of FGTB patients and HCW, respectively. The IFN-γ (+874T/A) polymorphism (A allele) was significant in 74.3 and 71.0% of FGTB patients and HCW, respectively, while the odds ratios for the AA and TA genotypes for predisposition of FGTB were found to be 0.304 and 1.650 in HCW, respectively. The SNP of TLR2 was not associated with FGTB but the SNP of IFN-γ was found to be associated with mycobacteria infections and to induce infertility.

**Conclusions/Significance:**

At present, we hypothesize that infertile women with FGTB and HCW without tuberculosis (TB) have identical frequency of TLR variants, which may be adequate in the production of IFN-γ in response to *Mycobacterium tuberculosis* infections. Thus, the study appears to be the first of its kind reporting a mutation in the IFN-γ gene [+874 T (thymine) to A] responsible for susceptibility to TB infections and further inducing infertility.

## Introduction

Female genital tuberculosis (FGTB) is usually an asymptomatic disease diagnosed during investigations for infertility [1–3]. Genital tuberculosis (GTB) represents 15–20% of extra pulmonary tuberculosis (EPTB) [4]. The incidence of infertility in GTB worldwide varies from 10–85% [5, 6]; it is endemic in India, with an incidence of 58% [7] and majority are in the reproductive age group (15–45 years) [8]. In 80–90% cases, it affects women with menstrual irregularities accounting for about 27% of manifestations of FGTB, [9] even this rate can be higher among patients with tubal factor infertility (39–41%) [10]. *Mycobacterium tuberculosis* (*M*. *tuberculosis*) is a facultative intracellular acid-fast gram-positive pathogenic bacterium capable of producing both a progressive disease and an asymptomatic latent infection [11, 12]. It generates disturbing effects by causing irreversible damage to the fallopian tube consequential in infertility which is hard to treatment both by medical and surgical methods [13, 14]. The exact cause as to why only some of those exposed to *M*. *tuberculosis* (MTB) develop uncontrolled, asymptomatic latent infection [12] and others eradicate or limit the disease remains unknown. Evidence suggests that the occurrence of tuberculosis (TB) at different rates among particular races, ethnicities, families, age group, gender and geographic area are majorly explained due to potential strains of microbial agents, defective host genetic factors and environmental factors [15–18]. These factors play a critical role in the susceptibility of *M*. *tuberculosis* infection, and, subsequently, in the development of infertility. Alterations in the cellular, molecular, biochemical, immunological, physiological, socio-behavioural functions of host have been partially elucidated due to genetic defects, low dietary value food, horrible life style (smoking), poor economical status and health care systems. Later on, play an important role in the development of progressive disease and indicating a genetic predisposition to TB susceptibility [15–22]. Ethiopathogenesis and immunopathogenesis of the disease engages quite a few components of the immune system includes innate immune cells and the adaptive immune response factors, which are implicated in the defence mechanisms against MTB [23–27]. Molecular biology and immunological studies have resulted in identification of several functional single nucleotide polymorphisms (SNPs) modulating infectivity and differential clinical presentations exist for genes encoding several types of proteins [28]. Out of which, toll like receptor 2 (TLR2) and interferon-γ (IFN-γ) genes are concern challenges due to their role in immune system, reproductive physiology and basic pathology at multiple levels [29–32]. In addition, it has been reported that TLR2 signalling pathway contributes to IFN-γ production [33–37]. Primarily, TLR2 gene plays an important role in pathogen recognition or other inflammatory stimuli, initiating the signalling cascades and directing the interactions between the immune and reproductive systems [38–45]. Similarly, the functions of IFN-γ are more diverse than the induction of bactericidal functions, control and clearance of intracellular pathogens [46–48], and includes the stimulation of antigen presentation with the helper T-cell type 1 (Th1) cytokine IFN-γ through class I and class II major histocompatibility complex (MHC) molecules [49], the orchestration of leukocyte-endothelium interactions, the effects on cell proliferation and apoptosis, as well as stimulation, regulation, cyclic expression and repression patterns of a variety of genes and their functional significance remains obscure [50–52]. IFN-γ is implicated as a major mediator of uterine natural killer (NK) cell functions during fertilization, pregnancy [34, 53–58], and thus involved in diagnosis and treatment of female infertility [59]. Exactly which function is undermined in +874A (adenine) individuals have not been determined yet. Expression of TLR2 gene is genetically controlled and the patients heterozygous for guanine (G) 2258A had reduced production levels of IFN-γ suggesting that altered TLR2 responsiveness might contribute to the course of infections [60–63] and weakened TLR2-IFN-γ signalling have been in differential clinical presentations [42, 44, 64, 65]. Recent data suggests that SNPs of TLR2 and IFN-γ genes play an important role in susceptibility to TB among different populations and subsequently, in the development of infertility [28, 66–69]. In a fascinating study by Darville *et al*., the course of a genital tract infection by one of the most common bacterial infections was observed in transgenic mice lacking TLR2. In the TLR2 knockout mice, significantly lower levels of the inflammatory cytokines, tumour necrosis factor-alpha (TNF-α) and macrophage inflammatory protein 2 were measured in genital tract secretions. Further, resulted in decreased inflammatory cell infiltration, dilatation, hydrosalpinx within the fallopian tubes and progression in the pathology of TLR2 deficient animals [44, 65, 66, 70]. Among its many effects, IFN-γ has an important role in activating macrophages in vascular endothelium and promotes plasma extravasations [71] and enhancing the expression of MHC-II molecules, resulting in enhanced antigen presentation to T cells. It has been reported to inhibit the cells of the trophoblast lineage outgrowth and trophoblast cell invasion to be accelerated in mice with genetic deficiency in the IFN-γ or IFN-γ receptor [72]. Intriguingly, SNPs in the IFN-γ gene has been variably correlated with susceptibility to TB among different populations [70], interfere with IFN-γ production, associated with varied clinical presentations [73, 74], in the etiology of female infertility due to mycobacterial infections [68]. Pravica *et al*., 2000 noted a novel SNP of alleles T (thymine) to A, located at the +874 position located from the translation start site in the first intron of IFN-γ gene is related to high and low IFN-γ expression, respectively, which in turn influences the immune responses during the course of infections [75]. Recently, SNPs of TLR2 2258G/A (Arg753Gln) and IFN-γ (+874T/A) gene that leads to a decreased response of macrophages to microbial peptides associated with receptor hyporesponsiveness and attenuated immune response with the host [76–79] has been reported. TLR2 gene polymorphism results in an arginine (CGG) to a glutamine (CAG) substitution and the resulting genotypes, therefore, are arginine/arginine (GG), glutamine/glutamine (AA) and arginine/glutamine (AG). However, the determination of functional SNPs and biochemical phenotypes (e.g., differential expression and functions of innate immune molecules like receptors, secondary mediators, cytokines, etc., by the host) in the immunopathogenesis of FGTB is necessary, assessing the contributions and functional consequences of specific genetic variations (polymorphisms) in the human genome in respect of host susceptibility or resistance to TB remains a longstanding challenge of population genetics research, with many questions unanswered. Also, in the vast majority of implicated genes, the molecular functions of candidate gene polymorphisms have remained unknown. A majority of the clinical association results have not been validated by molecular studies to reveal an underlying mechanism that could buttress the clinical results. Therefore, the aim of the present study was to investigate the risk of common SNPs of TLR2 gene, specifically at 2258G/A (Arg753Gln) and in IFN-γ gene, specifically at +874T/A in correlation with the occurrence of infertile women with FGTB and healthy women as controls. Further, we report the detailed clinical information of infertile women with FGTB, including their demographic information, pathological findings, microbiological evidences and infertile diagnosis.

## Materials and Methods

### Ethical Statement

The study protocol was in compliance with the Declaration of Helsinki, approved by the Institutional Ethics Committee (IEC) of Maternal Health and Research Trust (MHRT) Hospital & Research Centre, Hyderabad, India and also by Human Ethical Committee constituted by the Osmania University (OU), Hyderabad, India. Informed consent was documented by the use of a written consent form approved by the IEC and signed by the subject or the subject’s legally authorized representative. Each subject was asked whether the subject wants documentation linking the subject with the research, and the subject's wishes were govern; that the research presents no more than minimal risk of harm to subjects and involves no procedures for which written consent is normally required outside of the research context including operative diagnosis of infertility and its symptoms. Adequate time was given to the patients for the decision-making. A copy was given to the person signing the form. Consent was written rather than verbal because this is most appropriate in urban Indian communities with limited illiteracy, and where many individuals harbor mistrust of verbal documentation. Participant written informed consent was recorded prior to entering a subject into a study and documented the consent process in the subject’s medical record or the participant’s research record.

### Study design

A prospective case—control study was set in the Zoology Modular Lab, Central Facilities for Research and Development (CFRD), OU, Hyderabad, India. During the period of our study (September 2006–January 2015), the samples from infertile women visiting the gynaecology clinics at two collaborating maternal health care centres in Hyderabad were analysed. Our study was conducted on large number of patients and the samples were collected using convenience sampling method. All patients met the definitive/confirmed **inclusion criterion** for selecting FGTB-suspected cases showing clinical symptoms such as infertility among the reproductive age group (18–40 years), pelvic pain, scanty menstruation with irregular periods, dysmenpeorrhoea, oligomenorrhoea, amenorrhoea, general malaise and menorrhagia leading to the abortions. Radiological findings may or may not be indicative of TB and laparoscopic findings indicated beaded appearance, presence of tubercles and/or thickened tubes, hydrosalpinx, peritubal/periovarian adhesions and tubo-ovarian mass without frank tubercles. Histopathological findings indicated chronic inflammation or lesions such as proliferative solid epithelioid granulomas or caseation, dense polymorphonuclear cells, lymphocytic infiltrations, giant or beaded cells, enlarged lymphoid cells and accumulation of plasma and spindle cells. Demonstration of tubercle bacilli in culture, Ziehl-Neelsen (Z–N) staining of menstrual blood fluids, pelvic aspirated fluids (PAFs), endometrial curetting, endometrial tissue biopsies (ETBs) and ovarian tissue biopsies (OTBs) are indicative of TB. **Exclusion criteria** were as follows: women above 40 years of age, symptoms suggesting pulmonary TB/extra-pulmonary TB except infertility, with normal abdominal and vaginal examinations, pregnant and nursing women, women with autoimmune disorders, pulmonary infections, human immunodeficiency virus (HIV) co-infection, inflammatory diseases like intrauterine hepatitis B virus (HBV) infections, women with diabetes, malnutrition and other medical disorders like hypertension, peritoneal adhesions due to previous abdominal surgery, infertility due to male factors and abnormality in ovulations. As, the polymorphism at Arg753Gln (arginine to glutamine substitution at residue 753) region of TLR2 gene or IFN-γ +874T/A gene plays an important role in developing such complications.

Information on the general, obstetric and gynaecological details, including family history, marital status, age at menarche, length of menstrual cycle, associated symptoms, duration and amount of blood loss, duration of infertility and socio-demographic details like social status, occupation, lifestyle, age, body mass index (BMI), limited information on dietary and nutritional factors were obtained. Assessment of IFN-γ levels or IFN-γ signalling responses between the FGTB patients and healthy women were not optional, as, the Indian government banned serological antibody tests in 2012, and both Standards for TB Care in India (STCI) and International Standards for TB Care (ISTC) discourage the use of the interferon-gamma release assays (IGRAs) for the diagnosis of active TB [80, 81]. However, tuberculin skin test and erythrocyte sedimentation rates (ESR) was performed. All subjects were HIV-negative, HBV-negative and negative for pulmonary TB on the basis of complete history and physical examinations (chest X-ray, lung plain X-ray and by appropriate tests, such as a tuberculin skin test). [82] Apart from routine examinations, laparoscopy and hysteroscopy were performed at infertility workup as and when needed. Details of hystero-laparoscopy findings were noted. Beaded appearance of tubes, frank tubercles on the uterus and pelvic mass in variable combination aroused a suspicion. Constitutional symptoms such as sweating, evening rise of temperature and weight loss were not major complaints, while local organ dysfunction manifested in amenorrhoea, omental adhesions and bilateral tubal blockage are seen on hysterosalpingographic studies. All samples collected were examined by haematoxylin and eosin (H & E) staining [83], Z–N staining for acid fast bacilli (AFB) [84], as well as culture on Löwenstein—Jensen (L–J) egg media [85] and the MTB-specific multi-gene/multi-primer polymerase chain reaction (PCR) method [86] by which FGTB was confirmed. MTB [American type of culture collection (ATCC) 35836] reference stain isolates provided by the Department of Microbiology, Nizam’s Institute of Medical Sciences (NIMS), Hyderabad, India, were used as controls in each assay. The diagnosis was made based on morphological [82] and molecular investigations.

### Processing of tissue biopsy

The specimens (such as ETBs, OTBs and PAFs) collected from the lesions over the endometrium, ovaries and pelvis were mixed with sterile normal saline, transported in sterile vials to the laboratory and processed as per standard protocols [86–88].

### Decontamination and concentration (D & C)

All specimens were decontaminated and concentrated by a modified hypertonic saline—sodium hydroxide (HS–SH) procedure [86, 89].

### Histopathological examination

**Fig 1:**
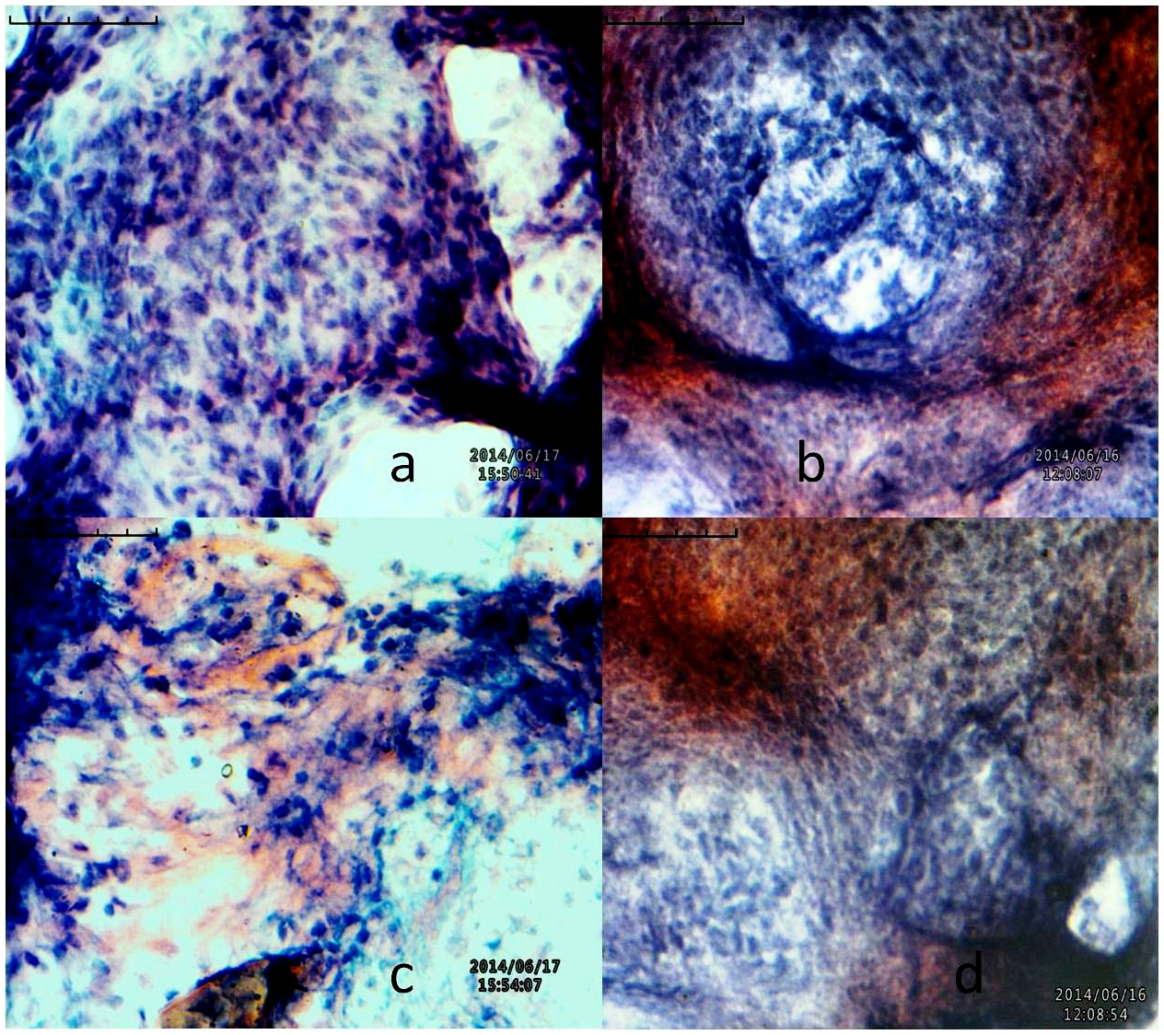
Histopathological examination of endo-ovarian tissue biopsy. a) polymorphonuclear cells were observed in endometrial tissue biopsies; b) Severe lesions with granulomatous filled with beaded and malignant lymphoid cells were observed in endo-ovarian tissue biopsies; c) appearance of spindle-cells with superficial strips of positive endometrial glands and stroma; d) lymphocytic infiltrations and initial stages of granulomatous were observed. **Note**: The microscopic studies were carried out with tissue biopsies and aspirated fluids containing tissue pieces. Thereafter, the slides were viewed under bright field (40X), Inverted microscope. The contrasts of the photographs are changed to improve the visibility.

Thin slices of the processed endo-ovarian tissue biopsies and PAFs were placed onto the slides and kept for air drying at room temperature. Specimens were fixed, dehydrated, cleaned and stained with Weigert’s iron H & E [83, 84, 86]. Then, the slides were cleaned and mounted. Mounted slides were viewed under a bright field (40X), Inverted Biological Microscope (BLM-290, BestScope, China). The presence of caseating granulomas surrounded by epithelioid cells, malignant lymphocytic infiltrations, plasma cells and giant polymorphonuclear cells were diagnostic of FGTB (Fig 1).

### Z–N staining of tissue sediments for AFB

**Fig 2:**
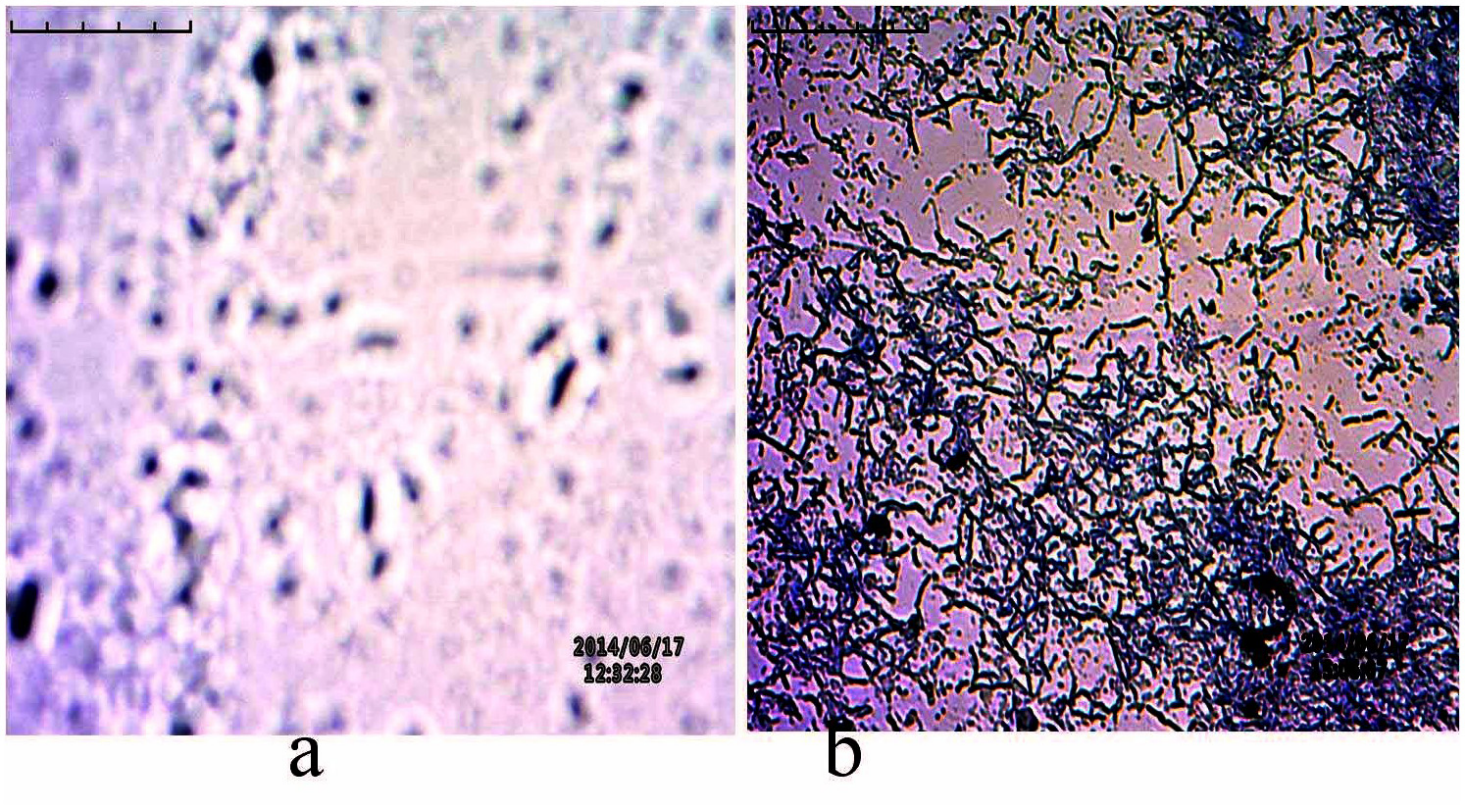
Z-N staining of endo-ovarian tissue biopsy and cultures in the detection of AFB. a) Red/ pink colour rod like beaded structures were observed in the tissue biopsy; b) Red/ pink colour rod like structures were observed on pale blue background in the cultures.

Stained slides were viewed under an Inverted Biological Microscope. The portion of smear that stained pink/red on a pale blue background was noted as *Mycobacterium* [84–86] (shown in Fig 2).

### Culture of tissue sediments

Inoculated culture tubes were incubated at 37°C for 6–8 weeks until heavy growth was obtained [86]. Cultures were stained by Z–N staining for confirmation of AFB growth [85]. “Standard Precautions” [90] and institutional guidelines have been followed in handling all items contaminated with blood and other body fluids.

### Quality control

In present study, we report the evidence based observatory facts gathered by means of different experiments. All these investigations have been conducted meticulously with suitable controls, standard strains; positive controls, negative controls (distilled water) and large sample size were correlated with different experimental parameters. Technical considerations, such as use of replications and retesting of doubtful positive samples were considered to influence the sensitivity and specificity. Therefore, all necessary measurements were taken to make the study technically sound. Reagents were aliquoted and each aliquote was used only once. Sterile microcentrifuge tubes and PCR tubes were used for the PCR assay. Reagent preparation, deoxyribonucleic acid (DNA) extraction, DNA amplification and detection were performed in separate rooms to avoid cross-contamination of amplicons. The recommendations and regulations of the Clinical and Laboratory Standards Institute (CLSI)/ National Committee for Clinical Laboratory Standards (NCCLS) [90] were followed for quality control and standards.

### DNA Extraction and Purification

The genomic DNA was extracted from different tissue sources, such as ETB, OTB and PAFs using the DNASure Tissue Mini Kit method (Genetix Biotech Asia Private Limited, New Delhi, India). Lysis was achieved by incubation of the sample material with Proteinase K solution in dry bath at 56°C (degree Celsius) for 1 to 3 hours. Then, appropriate condition for binding of DNA to the silica membrane in the DNASure Tissue Mini Kit Columns was achieved by the addition of chaotropic salts and ethanol to the lysate. The binding process is reversible and specific to nucleic acids. Contaminations were removed by subsequent washing with two different buffers according to manufacturer instructions. Pure genomic DNA was finally eluted under low ionic strength conditions in a slightly alkaline elution buffer. The purity of DNA was checked on 0.8% agarose (Amresco, Amresco LLC, United States of America) gel electrophoresis, incorporated with ethidium bromide. The electrophoresis was carried out with 1X tris-acetic acid-ethylene diamine tetra acetic acid (EDTA) buffer (pH-7.4) at a constant voltage (110 V) for one hour. The bands in the gel were visualized and quantified under ultraviolet illumination (Gel Doc-XRT system, Bio-Rad, Hercules, California, United States of America).

### Amplification refractory mutation system-multi gene/ multi primer polymerase chain reaction (ARMS-MG/MP-PCR) method

TLR2 polymorphism at position 2258 of the open reading frame (G versus A) [62, 77, 91] and IFN-γ polymorphism at position +874 in the first intron (T versus A) [75, 92] were identified by ARMS-MG/MP-PCR method using seven primers in a single tube- single step reaction. Primer selections for ARMS-MG/MP-PCR study were done based on their importance in the reproductive physiology, immunopathogenesis and progression of the disease. The nucleotide sequences of the primers used to amplify the TLR2 2258G/A (Arg753Gln) and IFN-γ +874T/A gene were described in the past ([62, 75, 77, 91, 92]; Table 1).

**Table 1:** Primers used for the detection of polymorphism among TLR2 2258G/A (Arg753Gln) and IFN-γ (+874T/A) genes.

Gene and Allele	Primer and length in base pairs (bp)	Sequence	Amplicon size (bp)	Annealing temperature	Reference
**Reaction 1**					
**TLR2 allele "G"**	TLR2 F (20)	5’ TATGGTCCAGGAGCTGGAGA 3’	470 & 328	62, 56	62, 77, 91, 93–96
	TLR2 R (25)	5’ TGACATAAAGATCCCAACTAGACAA 3’	470	62, 56	62, 77, 93–96
	TLR2 G (23)	5’ GGTCTTGGTGTTCATTATCTTCC 3’	328	62, 56	62, 77, 93–96
**IFN-γ allele "T"**	IFNG +874T F (24)	5’ TTCTTACAACACAAAATCAAATCT 3’	261	62, 56	75, 92
	IFNG +874A/T R (20)	5’ TCAACAAAGCTGATACTCCA 3’	261	62, 56	75, 92
**Human β-Globin gene (Internal control)**	HBG FWD (20)	5’ ACACAACTGTGTTCACTAGC 3’	110	56	75, 92
	HBG REV (20)	5’ CAACTTCATCCACGTTCACC 3’	110	56	75, 92
**Reaction 2**					
**TLR2 allele "A"**	TLR2 F (20)	5’ TATGGTCCAGGAGCTGGAGA 3’	470 & 328	62, 56	62, 77, 91, 93–96
	TLR2 R (25)	5’ TGACATAAAGATCCCAACTAGACAA 3’	470	62, 56	62, 77, 93–96
	TLR2 A (23)	5’ GGTCTTGGTGTTCATTATCTTCT 3’	328	62, 56	62, 77, 93–96
**IFN-γ allele "A"**	IFNG +874A F (24)	5’ TTCTTACAACACAAAATCAAATCA 3’	261	62, 56	75, 92
	IFNG +874A/T R (20)	5’ TCAACAAAGCTGATACTCCA 3’	261	62, 56	75, 92
**Human β-Globin gene (Internal control)**	HBG FWD (20)	5’ ACACAACTGTGTTCACTAGC 3’	110	56	75, 92
	HBG REV (20)	5’ CAACTTCATCCACGTTCACC 3’	110	56	75, 92

T: thymine; A: adenine; G: guanine; C: cytosine.

**Fig 3:**
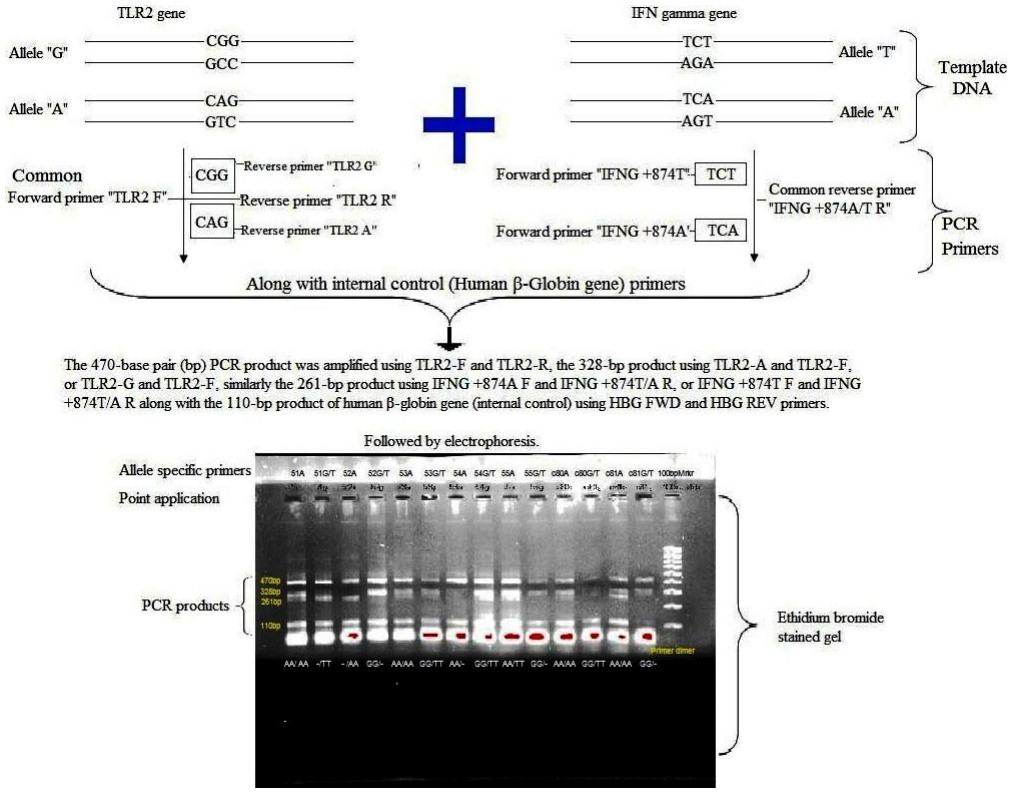
Amplification refractory mutation system-multi gene/ multi primer polymerase chain reaction (ARMS-MG/MP-PCR) method. ARMS-MG/MP-PCR method genotyping assay for detection of G to A nucleotide substitution for TLR2 and T to A substitution for IFN-γ gene. Results for the three possible genotypes (GG, AG and AA) for TLR2 and IFN-γ gene (TT, TA, AA) are shown above the PCR products. Three percent agarose gel electrophoresis was carried out with ARMS-MG/MP-PCR products: Lanes 51a to 55g were loaded with ARMS-MG/MP-PCR products of female genital tuberculosis (FGTB) patients. Lanes C80a to C81g was loaded with PCR products of healthy control women (HCW) without tuberculosis (TB). Lane 100 bp Mrkr was loaded with 100 base pair (bp) molecular weight marker (the 100bp size of product starts from the bottom side of the gel and ends with 1000 bp of product on the top/upper side of the gel). The upper bands symbolize the 470 bp internal control PCR fragment, the 328 bp bands represent the ‘**A**’ (lane 51a) and ‘**G**’ (lane 55g) alleles of TLR2 (Arg753Gln) gene. The 261 bp bands represent the ‘**A**’ (lane 51a) and ‘**T**’ (lane 55g) alleles of IFN-γ (+874) gene, and lower bands stands for the human β-globin gene (110 bp internal control). Primer dimers were also noted at the bottom, during the end of the sample run.

ARMS-MG/MP-PCR was performed with 20–30 ng (nanogram) extracted genomic DNA, and DNA amplification with 3 Units (U) of *Thermus aquaticus* (Taq) DNA polymerase (Bangalore Genie, Bangalore, India), 10 mM (millimolar) deoxyribonucleoside triphosphates (Bangalore Genie, Bangalore, India) and 13.5 pmol (picomol) each primer (Bioserve Biotechnologies Private Limited, Hyderabad, Telangana State, India). The PCR reaction was carried out for amplifying the genes by using two different set of reactions. The reaction for the common allele **(G)** was performed with TLR2-F (forward), TLR2-R (reverse) and TLR2-G primers along with the reaction for allele **“T”** of IFN-γ gene with IFNG +874T F (forward) and IFNG +874T/A R (reverse) primers. Another set of reaction was performed for the rare allele **(A)**, with TLR2-F, TLR2-R and TLR2-A along with the reaction for allele **“A”** of IFN-γ gene with IFNG +874A F and IFNG +874T/A R (Bioserve Biotechnologies Private Limited, Hyderabad, Telangana State, India) in a final volume of 50 μL (micro litters) along with internal control (human β [beta]-globin gene) with the following conditions. Two looped touchdown ARMS-MG/MP-PCR programmes, each with 25 cycles, were followed. In the first loop, the template DNA was initially denatured at 95°C for 5 min (minutes) and then denatured at 94°C for 45 s (seconds), annealed at 62°C for 45 s, extended at 72°C for 45 s and continued for a total of 25 cycles. In the second loop, DNA was denatured at 94°C for 45 s, annealed at 56°C for 45 s, extended at 72°C for 45 s and continued for a total of 25 cycles with a final extension at 72°C for 15 min. The PCR amplification was done using the Mastercycler gradient PCR system (Eppendorf, Hamburg, Germany). PCR products were subjected to electrophoresis on a 3% (percent) agarose gel incorporated with ethidium bromide, along with Gene Rule 50-base pairs (bp) and 100-bp DNA ladder/molecular weight marker. The electrophoresis was carried out with 1X tris-acetic acid-EDTA buffer (pH-7.4), at a constant voltage (110 V) for one hour. The bands in the gel were visualized under ultraviolet illumination (Gel Doc-XRT with molecular image lab software, Bio-Rad, Hercules, California, United States of America). As, we found clear and accurate banding patterns by agarose gel electrophoresis, sequencing of the PCR product was not suggested. The results of FGTB cases were compared with healthy control women (HCW) as shown in Fig 3. The 470-bp PCR product was amplified using TLR2-F and TLR2-R, the 328-bp product using TLR2-A and TLR2-F, or TLR2-G and TLR2-F, further the 261-bp product using IFNG +874A F and IFNG +874T/A R, or IFNG +874T F and IFNG +874T/A R along with the 110-bp product of human β-globin gene (internal control) using HBG FWD (forward) and HBG REV (reverse) primers.

### ARMS-MG/MP-PCR-Restriction Fragment Length Polymorphism (RFLP) for TLR2 2258G/A (Arg753Gln) and IFN-γ (+874T/A) gene

Screening for the TLR2 2258G/A (Arg753Gln) and IFN-γ (+874T/A) gene was performed using ARMS-MG/MP-PCR, later on analysed by RFLP [77, 93–95]. 15μL ARMS-MG/MP-PCR product containing TLR2 (2258G/A), IFN-γ (+874T/A) and β-globin gene were digested at 37°C for two hour with 1μL (10 U) PstI restriction enzyme (FastDigest Enzyme, Fermentas, United States of America) in a single tube reaction, respectively. Two reactions included with both genes; one for the common alleles **(G/T)** and another for the rare alleles **(A/A)** were performed. Positive control for complete digestion and negative controls for partial digestion were used to analyse the polymorphism among case-control groups [96]. After enzymatic digestion in 1X FastDigest buffer (Fermentas, United States of America), the samples were heat inactivated at 70°C for 5 minutes. Then, the fragments were subjected to electrophoresis on a 3% agarose gel (Amresco LLC, United States of America) incorporated with ethidium bromide, along with Gene Rule 50 bp DNA ladder/ molecular weight marker. The electrophoresis was carried out with 1X tris-acetic acid-EDTA buffer (pH-7.4) at a constant voltage (110 V) for one hour. The bands in the gel were visualized under ultraviolet illumination (Gel Doc-XRT with molecular image lab software, Bio-Rad, Hercules, California, United States of America). As, we find clear and accurate banding patterns by agarose gel electrophoresis, sequencing of the PCR products were not the optional. The results of different experiments were compared within case-control groups.

### Statistical analysis

The association between various quantitative variables (age, age at menarche, BMI and duration of infertility among infertile women with FGTB and HCW) were analysed by using SPSS v 20 for Windows (IBM SPSS Statistics 20, Chicago, United States of America). The data was presented as mean ± standard deviation (SD). The frequencies of the marker alleles were estimated by allele counting method. Association among qualitative variables (findings of conventional versus molecular methods, allele frequencies and genotype distribution among infertile women with FGTB and HCW) were statistically analysed by Pearson’s Chi-squared (χ2) test or Fisher’s exact test or McNemar’s test or Mantel-Haenszel common odds ratio (95% confidence interval [CI]), as needed. The significant differences in the positive rates of different methods were analysed. Departure from Hardy-Weinberg Equilibrium (HWE) test among FGTB patients and HCW were calculated [97]. The odds ratio (OR), degree of freedom (df) and *p*-values were used to measure the strength of the association among genotypes, FGTB and HCW. Data were considered statistically significant if *p*-value was less than 0.05.

## Results

Present study conducted a gene polymorphism analysis of TLR2 (2258G/A) and IFN-γ (+874T/A) genes, for their effect on infertility in women with FGTB and healthy women as controls. We selected a set of SNPs from the TLR2 and INF-γ genes to tag the common variation within these genes as well as to test functional variants using ETBs, OTBs and PAFs as the samples. A total of 302 specimens were taken for study which included samples from 202 infertile women having genital tuberculosis together with samples from 100 healthy control women (without TB) of reproductive age. Majority of the FGTB patients and HCW were ethnically of Indian origin and living in South India and in its states. We present detailed clinical information of this cohort, including their demographic information (Table 2).

**Table 2:** Association of demographic findings among infertile women with female genital tuberculosis (FGTB) and healthy control women (HCW) without tuberculosis (TB).

Variables	FGTB cases (N = 202)	HCW without TB (N = 100)	F-test values by ANOVA	Test of significant (by ANOVA)
**Age (Years)**	28.54± 4.46	27.59± 4.62	2.894	0.090
**Age at menarche (Years)**	12.49± 1.02	12.37± 0.93	0.965	0.327
**Body mass index (kg/m2)**	24.36± 1.47	24.05± 1.68	2.538	0.112
**Duration of infertility (Years)**	3.92± 3.03	0.174± 0.184	151.653	0.000

**Note**: Data was represented as mean± standard deviation (SD). Data were considered statistically significant if *p*-value was less than 0.05. The degree of freedom (df) was one (1) for all calculations. kg: kilogram. m: meters. N: number of patients. ANOVA: analysis of variance.

The mean age of the subjects was 28.54±4.46 years, mean duration of infertility was 3.92±3.03 years and BMI was 24.36±1.47. Statistical association was observed in relation with different parameters among case—control groups (F-test = 151.653, 1 df, p <0.0001). Subsequently, we undertake an association study in a well-characterized set of infertile patients (N = 175) confirmed as having FGTB [98] and healthy control women (N = 100) without TB. Out of 275 specimens collected from 275 case-control groups, 134 (48.72%) were premenstrual ETBs, 85 (30.9%) were OTBs and 56 (20.36%) were PAFs. Statistical analysis of tissue biopsies explained the intensity of pathological condition in association with different sites and its infectivity (Pearson’s χ2 = 27.765, 1 df, Fisher’s exact test value <0.0001, McNemar’s test value <0.021). Similarly, tissue biopsies were useful in examining the significant association with different clinical outcomes such as infertility, abortions and etc.

**Fig 4:**
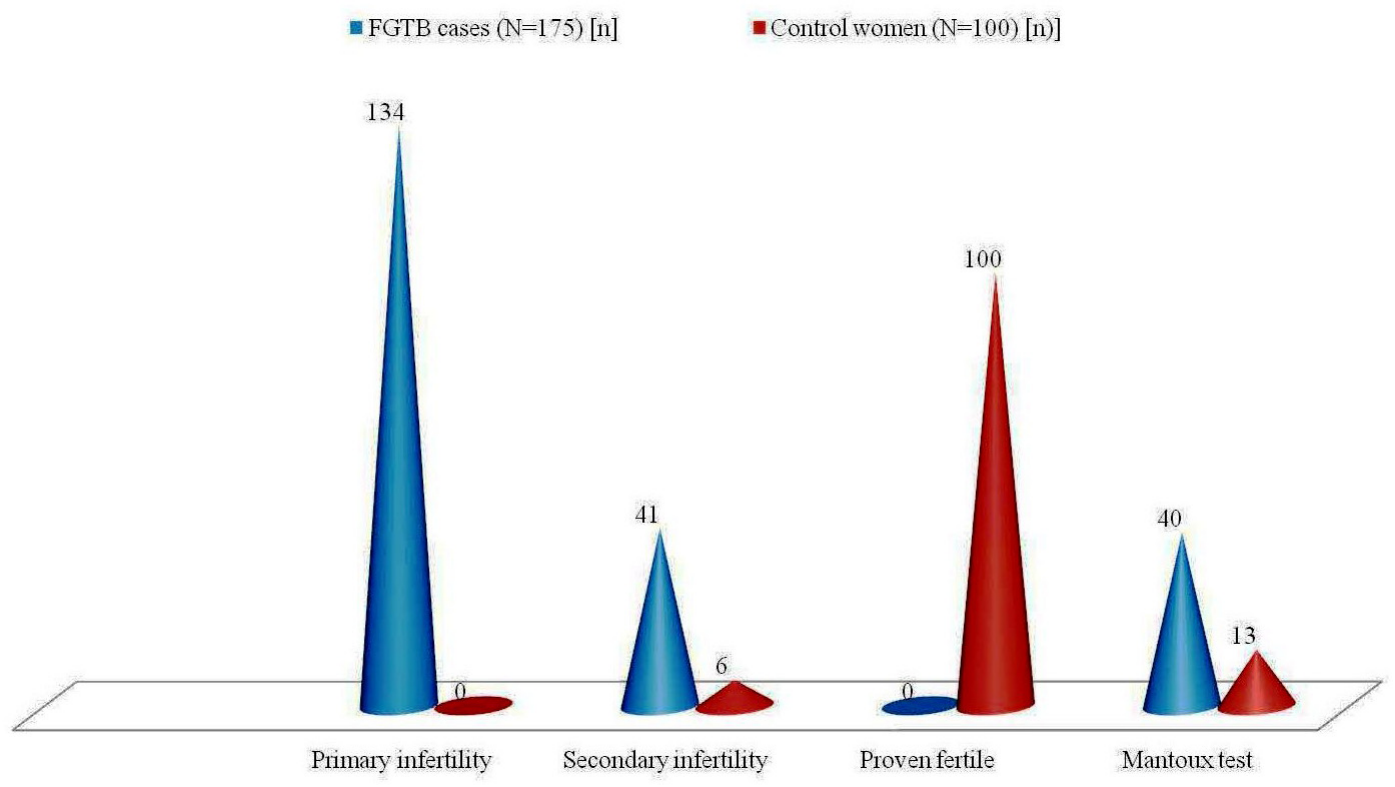
Association of infertility and Mantoux test among infertile women with female genital tuberculosis (FGTB) and healthy control women (HCW) without TB.

Table 3 partly explains the statistical association of different tissue biopsies (site specific pathology) among case-control groups. Of the 175 FGTB patients, majority of them (76.6%) presented primary infertility, 41 (23.4%) with secondary infertility (Fig 4) and 59 (33.7%) women experienced abortion.

**Table 3:** Statistical association of different tissue biopsies (site specific pathology) among case-control groups.

Types of samples	Infertile women with TB (N = 175) [n (%)]	Control women without TB (N = 100) [n (%)]	Pearson’s χ2 values	Pearson’s χ2 asymptotic significance (two-sided) values	Two sided Fisher's exact test values	Two sided McNemar’s test^d^ values
**Endometrial tissue biopsy**	107 (61.1)	27 (27)	27.765^a^	0.000	0.000	0.021
**Ovarian tissue biopsy**	58 (33.1)	27 (27)	1.124^b^	0.289	0.343	0.221
**Pelvic aspirated fluids**	10 (5.7)	46 (46)	63.686^c^	0.000	0.000	0.000

**Note**: Statistical analysis showed 0 cells (0.0%) have an expected count less than 5 for all methods. The minimum expected count (MEC) for different methods were different, as the MEC for “a” is 48.01; “b” is 30.91 and “c” is 20.36. “d” denotes the use of a binomial distribution. Degree of freedom (df) was 1 (one) for all calculations. Data were considered statistically significant if *p*-value was less than 0.05. N: number of patients; n: number of samples; %: percentage.

Apart from infertility, 109 (62.3%) patients had other menstrual complaints such as dysmenorrhoea in 84 (48%) women and abdominal/ pelvic pain in 25 (14.3%) women. Fifty two (29.7%) patients had menstrual irregularities such as oligomenorrhea (10.9%), amenorrhea (9.1%), general malaise (5.1%) and menorrhagia (4.6%) among infertile women. Forty four (25.14%) women were found to be without dyspareunia and dysmenorrhoea.

Table 4 elucidates a greater frequency of menstrual complaints among infertile women with FGTB and HCW without TB. The statistical difference was significant, specified with Pearson’s χ^2^ value = 5.338, 1 df, Fisher’s exact test value <0.021 and χ^2^ value = 0.021, McNemar’s test value <0.0001. All FGTB patients were negative for chest X-ray and 40 (22.9%) patients were positive for Mantoux test. ESR was elevated in all FGTB patients, which showed the reading between 56 and 156. All the modalities of treatments such as ovarian induction, intra uterine injection, intra cytoplasmic sperm injection, in-vitro fertilization in 3^rd^-4^th^ cycles and appropriate surgical intrusions were unsuccessful to support fertility prior to anti-tubercular chemotherapy. Favourable infertility outcomes following the anti-tubercular chemotherapy was reported among FGTB cases [99]. Further, efforts were made to assess SNPs among infertile women with FGTB rather than the treatment. Laparoscopy usually detects macroscopic changes such as beaded tubes in 118 (67.4%) women, tubal block with hydrosalphinx in 101 (57.7%), tubercular salpingitis in 83 (47.4%), omental adhesions in 65 (37.1%) and multiple tubercules in 61 (34.9%) women were recorded in the case group [82]. A standard protocol of investigations revealed a number of causes for fertility deprivation. The clinico-pathology of infertile women with FGTB were significantly dissimilar from that of the HCW (Pearson’s χ^2^ = 9.524, 1 df, Fisher’s exact test value <0.002). The difference was due to over-representations of different clinical symptoms among infertile women with FGTB (Pearson’s χ^2^ = 118.107, 1 df, McNemar’s test value <0.250).

**Table 4:** Arithmetical association of menstrual problems among infertile women with FGTB and HCW without TB.

Symptoms	FGTB cases (N = 175) [n (%)]	HCW without TB (N = 100) [n (%)]	Pearson’s χ2 values	Pearson’s χ2 asymptotic significance (two-sided) values	Two sided Fisher's exact test values	Two sided McNemar’s test^a^ values
**Dysmenorrhoea**	84 (48)	0 (0)	69.110^b^	0.000	0.000	0.269
**Abdominal pain**	25 (14.3)	0 (0)	15.714^c^	0.000	0.000	0.000
**Oligomenorrhea**	19 (10.9)	3 (3)	5.338^d^	0.021	0.021	0.000
**Amenorrhea**	16 (9.1)	0 (0)	9.708^e^	0.002	0.001	0.000
**General malaise**	9 (5.1)	0 (0)	5.317^f^	0.021	0.029	0.000
**Menorrhagia**	8 (4.6)	7 (7)	0.728^g^	0.394	0.417	0.000
**Women with abortion**	59 (33.7)	0 (0)	42.923^h^	0.000	0.000	0.001

**Note**: Statistical analysis showed 0 cells (0.0%) have an expected count less than 5 among all methods, except for “f” 1 cell (25.0%) has expected count less than 5 and the MEC is 3.27. The MECs for different methods were different, as the MEC for “b” is 30.55; “c” is 9.09; “d” is 8.00; “e” is 5.82; “g” is 5.45 and “h” is 21.45. “a” denotes the use of a binomial distribution. Degree of freedom (df) was 1(one) for all calculations. Data were considered statistically significant if *p*-value was less than 0.05.

Table 5 reveals a greater frequency of macroscopic changes among infertile women with FGTB and HCW without TB. Of the 175 specimens taken (from 175 infertile women with FGTB) for study, 107 (61.1%) were ETBs, 58 (33.1%) were OTBs and 10 (5.7%) were PAFs. Women who attended the same clinic for other gynaecological disorders, tubal sterilisation and laparoscopy for menorrhagia were selected as controls. A total of 100 specimens were collected from 100 healthy control women of reproductive age (18–40 years). All the women in the control group were asymptomatic and were normal, with a mean age of 27.59 ± 4.62 years, the mean duration of infertility was 0.174 ± 0.184 years and the mean BMI was 24.05 ± 1.68 (Table 2). All HCW (negative for pulmonary TB) were fertile, negative for chest X-ray and laparoscopically confirmed to be without FGTB. Thirteen healthy women were found positive with the Mantoux test, which indicates the serological positivity of controls with TB (Fig 4). Menstrual irregularities such as oligomenorrhoea (3%) and mild menorrhagia (7%) were seen in the HCW (Table 4). Only thickened tubes and tubal adhesions were observed in 4 (4%) control women on laparoscopic examinations (Table 5). Of the 100 specimens comprising the HCW, 27 (27%) were ETBs, 27 (27%) were OTBs and 46 (46%) were PAFs. None of the patients in our study reported a family history of TB.

**Table 5:** Statistical association of laparoscopic findings among infertile women with FGTB and HCW without TB.

Clinical symptoms	FGTB cases (N = 175) [n (%)]	HCW without TB (N = 100) [n (%)]	Pearson’s χ2 values	Pearson’s χ2 asymptotic significance (two-sided) values	Two sided Fisher's exact test values	Two sided McNemar’s test^l^ values
**Tubal block with hydrosalphinx**	101 (57.7)	0 (0)	91.215^a^	0.000	0.000	1.000
**Tubo-ovarian mass**	51 (29.1)	0 (0)	35.778^b^	0.000	0.000	0.000
**Tubercular salphingitis**	83 (47.4)	0 (0)	67.932^c^	0.000	0.000	0.237
**Beaded tubes**	118 (67.4)	0 (0)	118.107^d^	0.000	0.000	0.250
**Bilateral opening of tubes**	43 (24.6)	0 (0)	29.126^e^	0.000	0.000	0.000
**Thicken tubes & Tubal adhesions**	29 (16.6)	4 (4)	9.524^f^	0.002	0.002	0.000
**Tubercules on uterus**	18 (10.3)	0 (0)	11.006^g^	0.001	0.000	0.000
**Omental adhesions**	65 (37.1)	0 (0)	48.639^h^	0.000	0.000	0.008
**Frozen pelvis**	50 (28.6)	0 (0)	34.921^i^	0.000	0.000	0.000
**Multiple tubercules**	61 (34.9)	0 (0)	44.793^j^	0.000	0.000	0.003
**Small ovaries and lower abdominal mass**	30 (17.1)	0 (0)	19.242^k^	0.000	0.000	0.000

**Note**: Statistical analysis showed 0 cells (0.0%) have an expected count less than 5 for all methods. The MEC for different methods were different, as the MEC for “a” is 36.73; “b” is 18.55; “c” is 30.18; “d” is 42.91; “e” is 15.64; “f” is 12.00; “g” is 6.55; “h” is 23.64; “i” is 18.18; “j” is 22.18 and “k” is 10.91; “l” denotes the use of a binomial distribution. Degree of freedom (df) was one (1) for all calculations. Data were considered statistically significant if *p*-value was less than 0.05.

Out of the 275 specimens collected, 91 (33.1%) specimens from infertile women and 1 (0.36%) HCW was positive on H & E staining. AFB was positive in 43 (15.63%) specimens collected from infertile women and 1 (0.36%) HCW was positive by cultivation on L–J egg medium. Out of 83 culture-positive cases, 80 (29.1%) FGTB cases were found to be AFB positive by Z–N staining. 175 (63.63%) specimens were positive for the 19-kDa antigen (131 bp) gene and TRC4 (173 bp) repetitive element by multi-gene/multi-primer PCR. Disparity in the detection of FGTB by Z–N staining for AFB-positive, histopathological evidence of TB infection, detection of mycobacteria using culture and multi-gene PCR among infertile women and HCW are shown in Table 6.

**Table 6:** Diagnosis and statistical significance of FGTB infection among infertile women and HCW without TB.

Methods/ Characteristics	FGTB cases (N = 175) [n (%)]	HCW without TB (N = 100) [n (%)]	Pearson’s χ2 values	Pearson’s χ2 asymptotic significance (two-sided) values	Two sided Fisher's exact test values	Two sided McNemar’s test^f^ values
**H & E Staining**	91 (52)	1 (1)	74.348^a^	0.000	0.000	0.612
**AFB +Ve on Z-N Staining of tissue sediments**	43 (24.6)	0 (0)	29.126^b^	0.000	0.000	0.000
**L-J Egg medium**	83 (47.4)	1 (1)	64.659^c^	0.000	0.000	0.266
**AFB +Ve on Z-N Staining of culture**	80 (45.7)	0 (0)	64.469^d^	0.000	0.000	0.157
**Molecular analysis**	175 (100)	0 (0)	275.000^e^	0.000	0.000	0.000

**Note**: Statistical analysis showed 0 cells (0.0%) have an expected count less than 5 for all methods. Variation was observed for different methods in terms of the minimum expected counts, i.e. MEC for “a” is 33.45; “b” is 15.64; “c” is 30.55; “d” is 29.09 and “e” is 36.36. The “f” denotes the use of a binomial distribution. Degree of freedom (df) was one (1) for all calculations. Data were considered statistically significant if *p*-value was less than 0.05. Molecular analysis includes polymerase chain reaction (PCR) for 19kDa (131bp), 19kDa antigen gene; TRC4 (173bp), TRC4 element. AFB +Ve, AFB positive.

Fisher’s exact test and McNemar’s test were applied among FGTB cases and the HCW to ascertain the TB infection rate using conventional (AFB, culture and histopathology) and molecular methods. The *p*-value was highly significant (Pearson’s χ^2^ = 275.000, 1 df, Fisher’s exact test value <0.0001, McNemar’s test value <0.0001) with multi-gene PCR, whereas moderate agreement (Pearson’s χ^2^ = 64.659, 1 df, Fisher’s exact test value <0.0001, McNemar’s test value = 0.266) was reported with culture and mild agreement (Pearson’s χ^2^ = 74.348, 1 df, Fisher’s exact test value <0.0001, McNemar’s test value = 0.612) was observed with histopathology. All FGTB cases that were negative by multi-gene PCR were infertile. All false negative samples evidenced by conventional and molecular methods were likely to represent as negative for TB as multi-gene PCR was repeatedly proven negative. In the non-TB control group, all the tests were negative for TB. All HCW were found to be negative for TB with ETBs, OTBs and PAFs. These results showed that the molecular method is more accurate than the conventional methods [86–88].

### TLR2 gene polymorphism

The genotype distribution of TLR2 polymorphism did not differ significantly between the FGTB patients and HCW in general [100]. The genotype distribution of 2258G/A polymorphism in infertile women with FGTB was more or less similar to that found in the HCW.

Table 7 shows the TLR variants did not differ significantly between FGTB and HCW. The TLR2 2258G/A (Arg753Gln) polymorphism for allele ‘A’ was found in 101 (57.7%) out of 175 FGTB patients (Fig 3), whereas dominant homozygous carriers of the A/A allele polymorphism comprised 45 (25.7%) of the FGTB patients. The TLR2 Arg753Gln A allele occurred in 58 (58%) control women, whereas 26 (26%) were dominant homozygous carriers of this polymorphism. Thirty two (32%) heterozygous carriers (GA) of polymorphism were reported in the FGTB patients and HCW. However, FGTB patients showed slight increase in frequency of GG genotype (42.3% versus 42.0%) in comparison with HCW. HWE test [97] χ^2^ values were 20.46 and 11.78 for the FGTB patients and HCW, respectively. Allele frequencies for G and A were found in the FGTB patients to be 0.58 and 0.42 respectively. Similar results were observed in the HCW without TB. Quite a few statistical tests were performed for the evaluation of differences in the ratios of the three genotypes between the cases-control groups, the AA genotype was unlikely associated with FGTB than the GA and GG genotypes (Pearson’s χ^2^ asymptotic significance (two-sided) value = 0.958, 1 df, Fisher’s exact test value <1.000, McNemar’s test value <0.010) were given in Table 7. The odds ratios for the AA and GA genotypes for predisposing to FGTB were 1.015 (OR), 95% (CI) = 0.579–1.779 and 1.000 (OR), 95% (CI) = 0.591–1.693) in control group, respectively. In addition, computations for risk estimations were made under dominant and recessive models, showed a significantly lower risk for AA infertile women with FGTB at 100% (OR = 0.985, 95% CI = 0.562–1.726; *P* = 1.000). Under recessive model, ORs showed significant protection for GG individuals against developing FGTB among infertile patients (OR = 1.011, 95% CI = 0.615–1.664; *P* = 1.000), indicating that the results were insignificant.

**Table 7:** The genotype distribution of the TLR2 (2258G/A) gene polymorphism among infertile women with FGTB and HCW without TB.

Variables/ Statistical methods		FGTB cases (N = 175)	HCW without TB (N = 100)	Pearson’s χ2 values	Pearson’s χ2 asymptotic significance (two-sided) values	Two sided Fisher's exact test values	Two sided McNemar’s test^d^ values	Odds ratio (95% CI)
**Genotypes**	GG n (%)	74 (42.3)	42 (42)	0.002^a^	0.963	1.000	0.191	0.988 (0.601–1.626)
	GA n (%)	56 (32)	32 (32)	0.000^b^	1.000	1.000	0.323	1.000 (0.591–1.693)
	AA n (%)	45 (25.7)	26 (26)	0.003^c^	0.958	1.000	0.010	1.015 (0.579–1.779)
**Allele frequencies**	G	0.58	0.58					
	A	0.42	0.42					
**HWE χ^2^ values**		20.46	11.78					
Dominant Model*	AA n (%)	45 (25.7)	26 (26)	0.000	1.000	1.000		0.985 (0.562–1.726)
	GA+GG n (%)	130 (74.3)	74 (74)	0.000	1.000	1.000		0.985 (0.562–1.726)
Recessive Model*	GA+AA n (%)	101 (57.7)	58 (58)	0.000	1.000	1.000		1.011 (0.615–1.664)
	GG n (%)	74 (42.3)	42 (42)	0.000	1.000	1.000		1.011 (0.615–1.664)

**Note**: Statistical analysis showed 0 cells (0.0%) have an expected count less than 5 for all methods. Variation was observed for different methods in terms of the minimum expected counts, i.e. MEC for “a” is 42.18; “b” is 32.00 and “c” is 25.82. The “d” is denotes the use of a binomial distribution. The odds ratio (95% CI) values of the genotypes were designated as the Mantel-Haenszel common odds ratio estimate and values of asymptotic 95% CI lower bound and upper bound in the bracket. The Mantel-Haenszel common odds ratio estimate is asymptotically normally distributed under the common odds ratio of 1.000 assumptions. So is the natural log of the estimate. Degree of freedom (df) was one (1) for all calculations. Data were considered statistically significant if *p*-value was less than 0.05. “*” represents the use of a 2x2 contingency table via http://vassarstats.net/index.html online tool for the estimation of risk from dominant and recessive models. A: adenine; G: guanine.

Further, the TLR2 gene polymorphism was characterised by RFLP technique. Briefly, a 470 bp TLR2 gene fragment of the 4q arm of the genomic DNA, which included the mutation site, was replicated using TLR2 F (20) primer and TLR2 R (25) primer as listed in Table 1. Pst I restriction enzyme was used to recognized the specific sequences (CTGCAG) present in the TLR2 gene. For the heterozygous type (GA), this could be generated when digested with Arg753Gln and three bands of 254 bp, 214 bp and 40 bp, where one chain was cut, and the others were not [95]. For the homozygous type (AA), G transited to A, Pst I identified the mutated site, and the mutated DNA was visible as a double band of 214 bp and 40 bp, whereas a single band of 254 bp was observed in the wild type (GG), as the site was not cut. In this study, only the wild type (GG) was detected and no other types. The results of TLR2 gene polymorphism was against the previous findings. The sequence analysis was performed blinded to the phenotype of the patients.

### IFN-γ gene polymorphism

The significant difference in the SNP of IFN-γ (+874T/A) gene among infertile women with FGTB and HCW without TB are given in Table 8.

**Table 8:** The genotype distribution of the IFN-γ (+874T/A) gene polymorphism among infertile women with FGTB and HCW without TB.

Variables/ Statistical methods		FGTB cases (N = 175)	HCW without TB (N = 100)	Pearson’s χ2 values	Pearson’s χ2 asymptotic significance (two-sided) values	Two sided Fisher's exact test values	Two sided McNemar’s test^d^ values	Odds ratio (95% CI)
**Genotypes**	TT n (%)	45 (25.7)	29 (29)	0.349^a^	0.555	0.574	0.020	1.180 (0.681–2.043)
	TA n (%)	87 (49.7)	62 (62)	3.869^b^	0.049	0.059	0.000	1.650 (1.000–2.723)
	AA n (%)	43 (24.6)	9 (9)	10.063^c^	0.002	0.001	0.000	0.304 (0.141–0.653)
**Allele frequencies**	T	0.51	0.6					
	A	0.49	0.4					
**HWE χ^2^ values**		0.01	8.51					
Dominant Model*	AA n (%)	43 (24.6)	9 (9)	10.06	0.001	0.002		3.293 (1.530–7.088)
	TA+TT n (%)	132 (75.4)	91 (91)	10.06	0.001	0.002		3.293 (1.530–7.088)
Recessive Model*	TA+AA n (%)	130 (74.3)	71 (71)	0.350	0.554	0.573		0.847 (0.489–1.467)
	TT n (%)	45 (25.7)	29 (29)	0.350	0.554	0.573		0.847 (0.489–1.467)

**Note**: Statistical analysis showed 0 cells (0.0%) have an expected count less than 5 for all methods. Variation was observed for different methods in terms of the minimum expected counts, i.e. MEC for “a” is 26.91; “b” is 45.82 and “c” is 18.91. The “d” is denotes the use of a binomial distribution. The odds ratio (95% CI) values of the genotypes were designated as the Mantel-Haenszel common odds ratio estimate and values of asymptotic 95% CI lower bound and upper bound in the bracket. The Mantel-Haenszel common odds ratio estimate is asymptotically normally distributed under the common odds ratio of 1.000 assumptions. So is the natural log of the estimate. Degree of freedom (df) was one (1) for all calculations. Data were considered statistically significant if *p*-value was less than 0.05. “*” represents the use of a 2x2 contingency table via http://vassarstats.net/index.html online tool for the estimation of risk from dominant and recessive models. A: adenine; T: thymine.

The IFN-γ (+874T/A) polymorphism A allele overrepresented in 130 (74.3%) of the 175 FGTB patients (Fig 3), whereas dominant homozygous carriers of the A/A allele polymorphism comprised 43 (24.6%) of the FGTB patients. The IFN-γ (+874T/A) A allele presented in 71 (71%) control women, whereas 9 (9%) were dominant homozygous carriers of this polymorphism [82]. Most of the FGTB patients and controls showed heterozygous (TA) genotype (49.7% and 62% respectively) which is associated with intermediate IFN-γ production. However, FGTB patients also showed decrease in frequency of TT genotype (25.7% versus 29%) in comparison with controls. An increasing number of studies have shown that SNPs located in the promoter or coding regions of cytokine genes result in differential cytokine secretion due to altered transcriptional activation. It may be possible that the different stimuli results in differential transcription of the same gene [101]. Distributions of the genotypes in case control groups were analysed by HWE test [97]. HWE test *X* ^2^ values were 0.01 and 8.51 for the FGTB patients and control women, respectively. Allele frequencies for T and A were found to be 0.51 and 0.49 in the FGTB patient group and 0.6 and 0.4 in the control group, respectively. Statistical analysis of results were performed for the evaluation of differences in the ratios of the three genotypes between the cases and control group, the AA genotype was significant associated with FGTB than the TA and TT genotypes (Pearson’s χ^2^ asymptotic significance (two-sided) value <0.002, 1 df, Fisher’s exact test value <0.001, McNemar’s test value <0.0001) were given in Table 8. The odds ratios for the AA and TA genotypes for predispose of FGTB were found to be 0.304 (95% CI = 0.141–0.653) and 1.650 (95% CI = 1.000–2.723) in HCW without TB, respectively. Genotype TT of IFN-γ (+874T/A) showed a protective effect, while genotype AA was associated with increased susceptibility to develop FGTB and infertility. Further, computations for risk estimates made under dominant and recessive model showed a significantly high risk for AA infertile women with FGTB at 0.1% (OR = 3.293, 95% CI = 1.530–7.088; *P* = 0.001). Under recessive model, ORs showed significantly high protection for GG individuals against developing infertile among FGTB patients (OR = 0.847, 95% CI = 0.489–1.467; *P* = 0.554), indicating that the results were significant. Our results are in the line with recent studies which have been reported an association between the +874A/T SNP in the first intron of the IFN-γ gene and pulmonary TB [102], suggesting that the TT genotype which is associated with lower IFN-γ production confers susceptibility to FGTB [92]. Therefore, higher levels of IFN-γ can lead to more effective cell-mediated immunity against *mycobacterium*. A SNP, T to A, located at position +874 in the first intron could influence IFN-γ production levels. The association of different genotypes at this position, with a low (AA), medium (AT) and high (TT) cytokine production has been shown in vitro [103]. This is the first study investigating the genetic association of polymorphisms in the IFN-γ (+874 T/A) gene with FGTB patients using ARMS-MG/MP-PCR methods. FGTB patients with other confounding factors such as duration of infertility, menstrual problems, macroscopic findings of laparoscopy and diagnostic findings of TB infections by different methods were found to be associated with the TLR2 2258G/A and IFN-γ +874(T/A) gene polymorphisms. While, factors such as BMI, age at menarche and type of sample were not found to be associated with the gene polymorphisms. The IFN-γ SNP differs in frequency between the groups but since IFN-γ +874(A/T) alleles are in HWE for the cases but not in controls, suggests genotyping errors. However, these are been neglected in present study due uncertainty in the detection of specific errors including allelic dropouts and false alleles [104].

## Discussion

FGTB is hormone dependent as well so 90% of cases involve women less than 40 years of age [105]. It is major cause of infertility and rarely diagnosed in developed countries. It often has low-grade symptoms with very few specific complaints. The pathogenicity and mechanism of host response to FGTB is very controversial and still unclear. It usually spread to genital site from three routes, including hematogenous, sexual transmission [106], lymphatic or adjacent viscera [14], while it most commonly affects the fallopian tubes (95–100%), followed by the endometrium (50–60%), ovaries (20–30%), cervix (5–15%), and vulva/vagina (1%) and the myometrium (2.5%). [107, 108] The infections utmost depends on the site of inflammation [109], especially the spread of the pathogen to reproductive organs leads to a variety of clinical conditions [107] such as pelvic pain, menstrual irregularities, menorrhagia, oligomenorrhea, dysmenorrhoea, failure of implantation, postmenopausal bleeding, beaded tubes, tubal blocks, hydrosalpinges, tubo ovarian mass, where TB has been proven by endometrial biopsy, culture and laparoscopic findings [109–112]. Even, foetal growth retardation was also noted in several studies due to TB [113–116]. The heterogeneity in the clinical expression of FGTB strongly suggests the involvement of genetic factors on its immune pathogenesis, through the impact on gene expression of cytokines, chemokines and lymphokines, which are implicated in the host immune response and reproductive system. In fact, fertility is profoundly affected, not only by systemic infections that directly or indirectly target the reproductive organs, but also by various illnesses, intrauterine growth restriction, pre-eclampsia, preterm birth, recurrent pregnancy loss, endometritis, endometriosis, polycystic ovaries syndrome, ovarian cancer, chronic inflammatory conditions originated due to genetic polymorphisms in various genes [61–63, 70, 102, 103, 117–122]. Despite the important role that the TLR2 and IFN-γ plays in immune defence and reproduction, little is known about gene polymorphism in infertile women with FGTB. A mutation of TLR2 specifically inhibited MTB-induced cytokine production; this inhibition was incomplete, thereby suggesting that besides TLR2, other genes may be involved [123]. Similarly, compared to wild-type, the TLR2 2258A was reported to have a significant decrease in NF-kB (nuclear transcription factor kappa-light-chain-enhancer of activated B cells) response against bacterial peptides in human embryonic kidney (HEK) 293 T cells transfected with wild-type or TLR2 2258G/A (guanine (G) to adenine (A) substitution at position 2258) constructs [77]. In this context, we proposed to conduct a gene polymorphism analysis of TLR2 (2258G/A) and IFN-γ (+874T/A) genes in 175 infertile women with FGTB and 100 healthy women as controls.

In present study, we found that the allelic frequency of TLR2 Arg753Gln polymorphism gene (A allele) was 58% (26% dominant homozygous and 32% heterozygous) in healthy South Indian control women without TB. Our results clearly indicates that the frequency of dominant homozygous AA genotype (25.7%) among FGTB cases were slightly lower than healthy controls (26%). The TLR2 gene polymorphism study suggest that risk of developing FGTB among infertile women was not significant with AA (1.015) and GA (1.000) genotypes, respectively. Further, an increase in the odds ratio of AA genotype (1.015) when compared to GG genotype (0.988) was observed. Thus, genotype GG of TLR2 2258G/A showed a protective effect, while genotype AA may not be associated with increased susceptibility to develop FGTB. This finding improbably suggests a limited role of the TLR2 gene and its polymorphisms in disease susceptibility. This is the first report to analyse polymorphism in the TLR2 and IFN-γ gene, seeking an association between MTB infection and susceptibility to develop infertility among women with FGTB and control women without TB in an endemic region in India. Our report could provide observatory practical information on the South Indian population for studies designed at exploring the putative relevance of TLR2 (Arg753Gln) SNPs not only in FGTB, but also in a range of other infectious and inflammatory diseases among infertile patients. Fig 3 shows one of the simplest embodiments where two amplifications are carried out, one using a primer specific for the normal gene, and a second using a primer specific for the mutant gene. Generally, this approach is very suitable for rapid low cost genotyping. However, while using ARMS-MG/MP-PCR the conditions need to be very carefully controlled for accuracy. Under carefully controlled conditions (annealing temperature, magnesium concentration etc.), amplification only takes place if the nucleotide at the 3' end of the PCR primer is complementary to the base at the mutation site, with a mismatch being “refractory” to amplification. If the 3' end of the primer is designed to be complementary to the normal gene, then PCR products should be formed when amplifying the normal gene but not genes with the mutation, and vice versa. There are numerous variations of this approach. Genotyping is on the basis of the presence or absence of PCR products when using PCR primers specific for either the normal or mutant allele. However, absence of a product may also be due to sub-optimal ARMS-MG/MP-PCR conditions or low DNA quantity or quality. Therefore, it is common to include additional PCR primers for amplification of a control gene in the reaction mix, typically an unrelated gene. Absence of the PCR products of the control gene suggests sub-optimal conditions. This approach will be put on practice, wherever it is useful and information of this method will be made available to the public through the online websites.

In the past, the TLR2 R753Q (arginine 753 glutamine) polymorphism has been described in association with susceptibility to several infectious diseases such as tuberculosis, lepromatous leprosy, Lyme disease, *Salmonella* infection and *Candida* sepsis [61, 62]. Several studies have shown that the 753Q variant has functional relevance for TLR2 signaling. It has previously been demonstrated that the TLR2 polymorphism results in a decrease in the ability of macrophages to respond to several bacterial peptides [77]. Bochud *et al*., reported that polymorphism of the TLR2 gene caused severe impairment of the macrophage response to MTB and *Mycobacterium leprae* in both animal and human studies [124]. Many functional and genetic studies concluded that SNPs in TLRs have the ability to both modify receptor function and increased susceptibility of the host to TB infection. So far no functional domains have been identified in human TLR2, but potential functions of the carboxy (C) terminus include dimerization to form TLR2 homodimers or heterodimers with downstream targets such as myeloid differentiation primary-response gene 88 (MyD88) other adaptor molecules. Since the mutation is located at the very C terminus of TLR2, it likely affects the signalling function of the molecule, rather than ligand binding. Some studies indicate that the C terminus of TLR2 is involved in signaling and that Arg753 is specifically, important for this receptor function. Reiling *et al*., found that TLR2 knockout mice showed decreased resistance to TB and increased risk of developing disease on high-dose exposure, but no difference between the case and controls were observed under natural low-dose airborne infectious conditions [78]. However, there is no direct evidence that this polymorphism causes a decrease in immune response specifically to MTB.

Hence, we were unlikely to provide evidence for an association between the TLR2 gene polymorphisms and its role in inducing infertility among MTB infected cases and controls without TB. Our study did not achieve major statistical differences in the genotypes or allele distributions between cases and controls, but these insights do not exclude the role of gene polymorphisms in determining susceptibility to develop infertility possibly, due to imperceptible scrutiny of frequency. This can also possibly due to the existence of ethnical (racial) divergences, geographical divergences, climatic and environmental changes, sex divergences, exposure to infectious agents is not clear and further investigations are needed [124, 125]. However, proportional similarity in the allele frequency of TLR2 gene and genotypic shift among FGTB patients (57.7%) and healthy controls (58%) were reported, perhaps due to ideal genotypic analysis. The variation from the HWE test in the FGTB patients could be caused due to true genetic association. Thus, TLR2 gene polymorphism may not be the factors influencing the disease towards susceptibility. But, the defects in the factors other than TLR2 may be responsible in infertility and inducing the latent TB among such patients. Considering the possible reasons for the deviation from the HWE test encountered in the control women, since genotyping for both FGTB patients and control groups were performed simultaneously by the same research scholar in the same laboratory, it is thought that the genotyping error rate is minimal. Since, both case and control populations were selected from the same source population, it is contended that the observed SNPs association with FGTB unlikely relates to a direct functional effects of this polymorphism or linkage disequilibrium with another functional variants of TLR2 gene.

Specifically, we speculate that the infertile women with *M*. *tuberculosis* infection may have normal levels of TLR2 expression, which may be sufficient to induce a robust local Th1-type host defense (e.g. IFN-γ production) against the infection. Thus, maintaining the defective TLR2-IFN-γ signalling in favour of infections may be due to genetic variation in IFN-γ, leads to susceptibility of TB infections and further induces infertility. The fluctuation in the production of IFN-γ and its role in pathogenesis have been explained by specific mutation in IFN-γ +874A/T gene [64, 75, 92]. IFN-γ +874(T/A) polymorphism lies within the transcription factor binding site of the NF-κB, which shows an allele-specific binding pattern. The mechanism by which the IFN-γ +874T/A allele influences the susceptibility to FGTB may depend on its role in the regulation of IFN-γ production. The T allele of IFN-γ +874T/A provides a binding site for the NF-κB, which is able to regulate IFN-γ expression [75]. Heterozygous carriers have an intermediate phenotype, suggesting that more subtle variation in the IFN-γ response pathway may underlie susceptibility to TB in outbreed human populations [126]. Contrary to previous studies, we demonstrated that IFN-γ polymorphisms are responsible for the increased prevalence of infertility among women with mycobacterial infections, are influenced by virulence factors, the ability and availability of MTB in the South Indian population. In current study, the allelic frequency of IFN-γ (+874 T/A) gene polymorphism (A allele) was 71% (9% dominant homozygous and 62% heterozygous) in HCW without TB. Our observation shows that the individuals with IFN-γ +874 AA genotypes are more prevalent in 43 (24.6%) infertile women associated with FGTB clinical findings such as beaded tubes, tubal block with hydrosalphinx, tubercular salphingitis, omental adhesions along with menstrual complaints. These findings were not identified in 9 (9%) control women without TB. This nature of disease may be due to genetic variations in the bacterial strain, extraordinary virulent nature of *mycobacterium* and host genetic background. The study also explains the relevance of TLR2 and IFN-γ genes in the context of varied clinical presentations with the site specific pathology of TB infection. This may partly describe the tissue-specific expression of gene polymorphisms [127–129] merely observed in infertile women with FGTB. Suggests that, the mutation studies can be carried out irrespective of sample types, because re-investigation of IFN-γ gene polymorphism on different type of tissue biopsies among case-controls were resulted in consistent conclusion [92] with several previous reports of TB patients, while, the SNP of TLR2 gene was against the previous findings [63]. In this connection, several studies are conducted depending upon the geographic locality, ethnicity of a population, and variations have been reported regarding the occurrence and frequency of extrapulmonary tuberculosis in the both sexes, with different age groups and different the organs [130]. Approximately, 30% of healthy individuals are reported to be developing latent infections [131]. The differences in transmissibility and virulence among MTB strains are related to the genetic background and different lineages with specific geographical regions of the organisms [132]. Further, the factors like poverty, homelessness, immuno-suppression, ill health, acquired immuno deficiency syndrome (AIDS), drug abuse, malnutrition, drug resistant TB, multidrug resistant TB, total drug resistant TB, family and personal history of TB, and a poorly functioning of national tuberculosis program and dismantling of public health infrastructure have significantly contributed to the worsening situation [133] in India. Animal infection models suggests that the haematogenous dissemination of infection occurs before the onset of T-cell mediated immunity [134] and supports the hypothesis that the ability of different strains of MTB to produce different clinical phenotypes, varies depending upon their interaction with the host innate and adaptive immune responses, but the relevance of these findings to human disease remains uncertain [135]. Nonetheless, it is possible that more common genetic variants such as promoter region polymorphisms that influence gene expression are associated with the certain diseases. Therefore this study may provide innovative solution for solving unanswered problems with low cost accessories and practices. Certainly, we suggest that the IFN-γ +874 (A/T) alleles polymorphism were significantly associated with tuberculous bacillus infectivity and strongly plays role as a genetic risk factor for the pathogenesis of FGTB in Indian women. It is also possible that low IFN-γ production may impair antimycobacterial response against FGTB infection, rendering these individuals more susceptible to tuberculous bacillus infection other than pulmonary TB. Although IFN-γ can overcome these phenomena in vitro, MTB can interfere with IFN-γ signaling and down regulate the transcription of IFN-γ inducible genes [136].

In conclusion, this study corroborates the unremarkable frequency of the TLR2 Arg753Gln polymorphism observed in other populations and significant difference in allele frequencies have been reported between ethnically diverse populations. The TLR variant is at almost identical frequency in both groups. The IFN-γ SNP differs in frequency between FGTB patients and HCW. It is unlikely that TLR2 (R753Q) polymorphism may be associated with susceptibility to FGTB, while IFN-γ genotype at position +874T to A is strongly associated with etiology of FGTB. The SNP of IFN-γ is not only related to FGTB in disease development but also adding to the susceptibility of infertility caused by MTB infections. The statistical distribution of polymorphism between case- control groups may be due to other reason like extended duration of infertility, polluted environment that they live in and nutritional behaviours. However, the heterogeneity in the small number of available studies limits the ability to draw conclusions. Hence, the validations of these findings in independent cohorts are needed to firmly establish the role of TLR2 gene polymorphism, organ dysfunctions and infertility as well as clinical severity of FGTB. Further studies using a larger cohort of infertile patients with FGTB are warranted to identify the risk factors and genetic susceptibility to FGTB. The present study supports the perception that genetics, immunology, environment and infection may be vital in the pathogenesis of FGTB among infertile patients. A final proof of their functional relevance requires further studies to determine their functional effect on the immune response after their stimulation with relevant ligands and/or their association with immune related traits in FGTB patients. Collectively, these results do not support a significant role for reported mutations in the TLR2 gene in the observed differential functional responses to TLR activation in patients with FGTB, compared with that in healthy controls without TB. Detection of such polymorphisms in tuberculosis patients would help in assessing the risk of developing active FGTB among asymptomatic infertile patients and in identifying candidates for chemoprophylaxis. Further, we also suggested that asymptomatic nature of the disease, accessibility of reproductive clinics, and elucidation of genes associated with virulence, pressure of susceptible factors, detection of intraspecies differences in genome sequences and gene expression studies should not be neglected during the description of FGTB. More research is necessary to correlate mutations in TLR2 and IFN-γ with functional consequences. It is therefore necessary to screen a variety of candidate genes, such as those for cytokines and members of the TLR2-IFN-γ mediated signalling pathway with positive IFN-γ/IL-12 feedback loop, to characterize the genetics of female genital tuberculosis susceptibility among infertile women.
